# Reconciling the Potentially Irreconcilable? Genotypic and Phenotypic Amoxicillin-Clavulanate Resistance in *Escherichia coli*

**DOI:** 10.1128/AAC.02026-19

**Published:** 2020-05-21

**Authors:** Timothy J. Davies, Nicole Stoesser, Anna E. Sheppard, Manal Abuoun, Philip Fowler, Jeremy Swann, T. Phuong Quan, David Griffiths, Alison Vaughan, Marcus Morgan, Hang T. T. Phan, Katie J. Jeffery, Monique Andersson, Matt J. Ellington, Oskar Ekelund, Neil Woodford, Amy J. Mathers, Robert A. Bonomo, Derrick W. Crook, Tim E. A. Peto, Muna F. Anjum, A. Sarah Walker

**Affiliations:** a Nuffield Department of Medicine, Oxford University, Oxford, United Kingdom; b National Institute for Health Research Health Protection Research Unit on Healthcare Associated Infections and Antimicrobial Resistance at the University of Oxford, Oxford, United Kingdom; c Oxford University Hospitals NHS Foundation Trust, Oxford, United Kingdom; d Bacteriology, Animal and Plant Health Agency, Surrey, United Kingdom; e NIHR Biomedical Research Centre, Oxford, United Kingdom; f Antimicrobial Resistance and Healthcare Associated Infections (AMRHAI) Reference Unit, National Infection Service, Public Health England, London, United Kingdom; g Department of Clinical Microbiology and the EUCAST Development Laboratory, Kronoberg Region, Central Hospital, Växjö, Sweden; h Division of Infectious Diseases and International Health, Department of Medicine, Clinical Microbiology, Department of Pathology, University of Virginia Health System, Charlottesville, Virginia, USA; i Louis Stokes Cleveland Veterans Affairs Medical Centre, Research Service, Cleveland, Ohio, USA; j Case Western Reserve University, Departments of Medicine, Biochemistry, Molecular Biology and Microbiology, Pharmacology, and Proteomics and Bioinformatics, CWRU-Cleveland VAMC Centre for Antimicrobial Resistance and Epidemiology (Case VA CARES), and Geriatric Research Education and Clinical Centers, Louis Stokes Cleveland Department of Veterans Affairs, Cleveland, Ohio, USA

**Keywords:** antibiotic resistance, antimicrobial combinations, beta-lactamase inhibitor, microbial genomics, susceptibility testing

## Abstract

Resistance to amoxicillin-clavulanate, a widely used beta-lactam/beta-lactamase inhibitor combination antibiotic, is rising globally, and yet susceptibility testing remains challenging. To test whether whole-genome sequencing (WGS) could provide a more reliable assessment of susceptibility than traditional methods, we predicted resistance from WGS for 976 Escherichia coli bloodstream infection isolates from Oxfordshire, United Kingdom, comparing against phenotypes from the BD Phoenix (calibrated against EUCAST guidelines). A total of 339/976 (35%) isolates were amoxicillin-clavulanate resistant. Predictions based solely on beta-lactamase presence/absence performed poorly (sensitivity, 23% [78/339]) but improved when genetic features associated with penicillinase hyperproduction (e.g., promoter mutations and copy number estimates) were considered (sensitivity, 82% [277/339]; *P* < 0.0001). Most discrepancies occurred in isolates with MICs within ±1 doubling dilution of the breakpoint. We investigated two potential causes: the phenotypic reference and the binary resistant/susceptible classification. We performed reference standard, replicated phenotyping in a random stratified subsample of 261/976 (27%) isolates using agar dilution, following both EUCAST and CLSI guidelines, which use different clavulanate concentrations. As well as disagreeing with each other, neither agar dilution phenotype aligned perfectly with genetic features. A random-effects model investigating associations between genetic features and MICs showed that some genetic features had small, variable and additive effects, resulting in variable resistance classification. Using model fixed-effects to predict MICs for the non-agar dilution isolates, predicted MICs were in essential agreement (±1 doubling dilution) with observed (BD Phoenix) MICs for 691/715 (97%) isolates. This suggests amoxicillin-clavulanate resistance in E. coli is quantitative, rather than qualitative, explaining the poorly reproducible binary (resistant/susceptible) phenotypes and suboptimal concordance between different phenotypic methods and with WGS-based predictions.

## Abstract (precis)

Resistance to amoxicillin-clavulanate, a widely used beta-lactam/beta-lactamase inhibitor combination antibiotic, is rising globally, and yet susceptibility testing remains challenging. To test whether whole-genome sequencing (WGS) could provide a more reliable assessment of susceptibility than traditional methods, we predicted resistance from WGS for 976 Escherichia coli bloodstream infection isolates from Oxfordshire, United Kingdom, comparing against phenotypes from the BD Phoenix (calibrated against EUCAST guidelines).

## INTRODUCTION

Rising amoxicillin-clavulanate resistance in Escherichia coli is a major health care challenge, with increasing incidence of resistant bloodstream infections (BSI) (1) threatening its utility as the most commonly used antibiotic in Europe (2). Consequently, many hospitals are considering broadening their first-line empirical antibiotics for common infections. However, significant uncertainty is created by observed differences between the two main assays for amoxicillin-clavulanate susceptibility in the classification of clinical samples (3). These differences are so large that increasing amoxicillin-clavulanate resistance was suggested to be primarily due to laboratories switching from US Clinical and Laboratory Standards Institute (CLSI) to European Committee on Antimicrobial Susceptibility Testing (EUCAST) guidelines (4). Recent work (5), however, suggests that changes in laboratory protocols are unlikely to account for the majority of the increase in resistance. Only one study has investigated whether there are underlying genetic causes for the ongoing rise in amoxicillin-clavulanate resistance (6) but found no evidence of clonal expansion of any specific amoxicillin-clavulanate-resistant strains. However, the genetic epidemiology of amoxicillin-clavulanate resistance mechanisms was not investigated.

In addition to its widespread clinical use, amoxicillin-clavulanate is a model for beta-lactam/beta-lactamase inhibitor (BL/BLI) combinations, which are the focus of renewed attention (7) due to the development of novel BL/BLIs with activity against highly drug-resistant organisms (8). EUCAST has recently published guidelines on setting breakpoints for BL/BLIs (9), but the inconsistencies seen in testing and clinically interpreting amoxicillin-clavulanate resistance likely extend to novel BL/BLIs (10).

One solution is to instead identify the genetic determinants characterizing resistance (resistance genotype) using whole-genome sequencing (WGS) (11). This approach may be particularly helpful for BL/BLI, since recent studies have suggested that traditional phenotyping is less accurate in isolates producing extended spectrum beta-lactamases (12). Rather than resistance being associated with the simple presence or absence of specific genes, previous studies have found that much amoxicillin-clavulanate resistance is likely attributable to mechanisms which increase the effective concentration of beta-lactamases (e.g., additive effects of multiple beta-lactamases [13], increasing gene expression [14], or modifying cell permeability [15]). Given the added complexity of both phenotype and genotype, studies using WGS to predict phenotypic resistance have either not included amoxicillin-clavulanate (16, 17), compared against only one set of breakpoints (18), or only tested small sets of preselected samples (19). Similar studies investigating other BL/BLIs, such as piperacillin-tazobactam, reported poor accuracy when predicting resistance from genotype (20).

We therefore investigated concordance between WGS-derived genotypes and amoxicillin-clavulanate susceptibility phenotypes in a large, unselected set of Oxfordshire E. coli BSI isolates from 2013 to 2015. We assessed whether extending the usual presence/absence genetic approach to include features that might increase beta-lactamase expression (copy number and promoter type) would improve concordance, and quantified the impact of particular genetic variants and testing guidelines (EUCAST and CLSI) on MICs.

## RESULTS

### Routine laboratory phenotypes and amoxicillin-clavulanate resistance genotypes.

Of the 1,039 E. coli BSI occurring between January 2013 and August 2015 in Oxfordshire, UK, 1,000 had at least one isolate stored by Oxford University Hospitals (OUH) NHS Foundation Trust microbiology laboratory. In most (992/1,000 [99%]) infections, only a single E. coli strain was isolated; however, two different E. coli strains were grown from culture in 8 cases, giving a total of 1,008 distinct E. coli isolates. Each of these isolates had linked antimicrobial susceptibility test (AST) data from the OUH NHS Foundation Trust microbiology laboratory using the BD Phoenix (Becton, Dickinson, and Company). All obtained isolates were sequenced, with 976/1,008 (97%) having WGS data meeting predetermined quality controls designed to identify mixtures and poor-quality sequences. Overall, these 976 isolates represented 968/1,039 (93%) E. coli BSI (see Fig. S1 in the supplemental material). A total of 339/976 (36%) had amoxicillin-clavulanate MICs of >8/2 mg/liter according to EUCAST breakpoints (see Table S1 in the supplemental material).

The collection was highly diverse, representing 152 different sequence types (STs). The most common was ST73 (161 [17%]) (Fig. S2), followed by ST131 (124 [13%]), which had the highest percentage of phenotypically resistant isolates (*n* = 74 [60%]) and was the only ST associated with amoxicillin-clavulanate resistance (chi-squared *P* < 0.0001 compared to *P* > 0.16 for all other STs).

The most common beta-lactam resistance mechanisms identified (using ARIBA (21) (default parameters) and tBLASTn/BLASTn (see Materials and Methods)) were acquired beta-lactamase genes, which were identified in 515/976 (53%) isolates. Most of these (448/515 [87%]) harbored only a single transmissible beta-lactamase gene. Among the 67 isolates with more than one beta-lactamase gene, the most common combination was *bla*_CTX-M-15_ and *bla*_OXA-1_ (*n* = 27; see Table S2B in the supplemental material). Overall *bla*_TEM_ was by far the most common mechanism identified (occurring in 427/976 [44%] isolates), followed by *bla*_CTX-M_ (*n* = 73 [7%]), *bla*_OXA_ (*n* = 62 [6%]), and *bla*_SHV_ (*n* = 23 [2%]) (see Fig. S2 and Table S2 in the supplemental material). For the 594 transmissible beta-lactamases identified, the median DNA copy number from mapping coverage was 2.23 (interquartile range = 1.73 to 3.31); 227 (38%) had >2.5-fold coverage (the threshold to predict resistance derived from receiver operating characteristic [ROC] analysis of isolates with only one beta-lactamase identified; see the supplementary methods and Fig. S3 in the supplemental material). Variant *bla*_TEM_ and *ampC* promoters considered to be associated with increased expression were identified in 49 (5%) and 20 (2%) isolates, respectively (see Table S3A and 3C in the supplemental material). A total of 31 (3%) isolates potentially had one nonfunctional porin, 22 of which also contained a beta-lactamase gene. However, no isolate had “functionally lost” both *ompC* and *ompF* (see Table S4).

### WGS-derived resistance prediction compared to routine phenotyping.

We compared two genetic resistance prediction algorithms for amoxicillin and amoxicillin-clavulanate (see Materials and Methods). The first, denoted the “basic” prediction algorithm, was analogous to common WGS-based resistance methods and only predicted resistance for isolates containing inhibitor-resistant beta-lactamase genes (e.g., *bla*_OXA-1_ and *bla*_TEM-30_). The second, denoted the “extended” prediction algorithm, additionally evaluated *bla*_TEM_ and *ampC* promoter mutations, estimates of the beta-lactamase gene DNA copy number, and porin loss-of-function mutations. Including the additional features (i.e., the “extended” approach) had little impact on our ability to identify ampicillin resistance but significantly improved amoxicillin-clavulanate resistance prediction (Table 1, WGS-based ampicillin resistance prediction sensitivity of 98% [basic] versus 96% [extended]; compared to WGS-based amoxicillin-clavulanate resistance prediction sensitivity of 23% [basic] versus 82% [extended], McNemar’s *P* < 0.0001). However, the increased sensitivity also came at the cost of modestly reduced specificity (Table 1). The overall categorical agreement of WGS-derived with observed phenotype increased from 712 (73%) to 868 (89%) when these extended genetic features were included.

**TABLE 1:** Performance of WGS-based prediction using both basic and extended algorithms

WGS-predicted phenotype	AST phenotype (no. of isolates)		%	
	S	R	Sensitivity	Specificity
Ampicillin: basic				
S	441	20	96	99
R	4	511		
				
Ampicillin: extended				
S	439	8	98	99
R	6	523		
				
Amoxicillin-clavulanate: basic				
S	634	261	23	100
R	3	78		
				
Amoxicillin-clavulanate: extended				
S	591	62	82	93
R	46	277		

**FIG 1:**
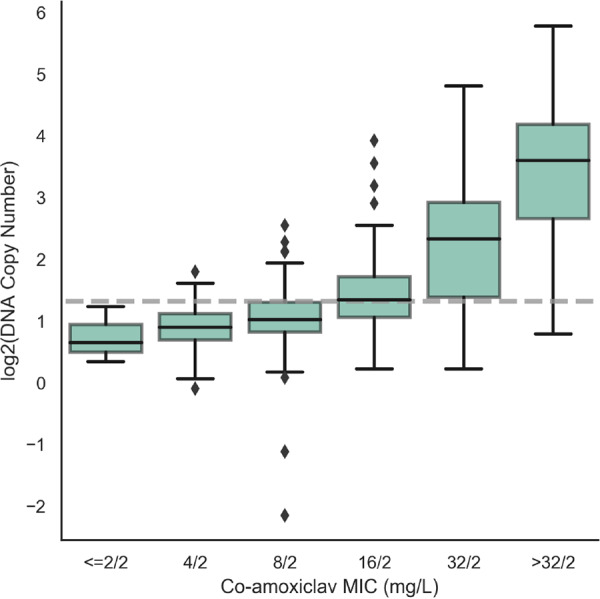
Association between transmissible beta-lactamase gene DNA copy number and amoxicillin-clavulanate MIC in isolates with no alternative resistance features. Evidence for the association between MIC and log_2_(DNA copy number) (*P* < 0.0001, estimated using quantile regression), is presented. The gray line indicates the 2.5 threshold used to define resistance in the extended algorithm based on ROC analysis (see Fig. S3A in the supplemental material). Of these 328 isolates, 294 had *bla*_TEM_ genes (290 with *bla*_TEM-1_, 4 with other non-inhibitor resistant *bla*_TEM_ genes), 19 had non-inhibitor-resistant *bla*_SHV_ genes, and 15 had *bla*_CTX-M_ genes.

Investigating the cause of lower than optimal agreement, even using the extended algorithm, showed that most false-positive predictions were made on the basis of increased beta-lactamase gene DNA copy number (Table 2). Although there was a clear association between increasing copy number and MIC (*P* < 0.0001), resistance prediction based on increased DNA copy number (>2.5) was less accurate than other extended algorithm components (positive predictive value [PPV] = 0.77 compared to >0.97 for all other algorithm components) with both resistant isolates with lower-copy-number beta-lactamases and susceptible isolates with higher-copy-number beta-lactamases (Fig. 1).

**TABLE 2:** Resistance prediction feature performance

Feature		Prediction	No. (%) of isolates resistant^*a*^	
			PPV	PPV in isolation^*b*^
Beta-lactamases				
	Any class C or D serine beta-lactamase	Basic	66/69 (96)	32/34 (94)
	Any inhibitor resistant class A beta-lactamase	Basic	12/12 (100)	2/2 (100)
			
Promoter mutations				
	Non-P3 *bla*_TEM_ promoter associated with *bla*_TEM_ hyper-production	Extended	48/49 (98)	29/30 (97)
	*ampC* promoter mutation associated with a*mpC* hyper-production	Extended	20/20 (100)	13/13 (100)
			
Increased DNA copy number				
	Relative coverage of any transmissible beta-lactamase > 2.5^*c*^	Extended	184/227 (81)	128/167 (77)
			
Decreased permeability^*d*^				
	Features suggesting disruption of either *ompC* or *ompF* in an isolate containing an additional beta-lactamase (see the supplementary methods)	Extended	19/22 (86)	2/2 (100)

a That is, the number determined to be resistant by routine AST/total isolates with this feature. PPV, positive predictive value.

b That is, the PPV restricted to isolates not predicted to be amoxicillin-clavulanate resistant by any other feature.

c Cutoff chosen following a receiver operating curve (ROC) analysis (see the supplemental material, Fig. S3A, in particular).

d Beta-lactam resistance features in isolates are presented in Tables S2, S3, and S4 in the supplemental material.

**FIG 2:**
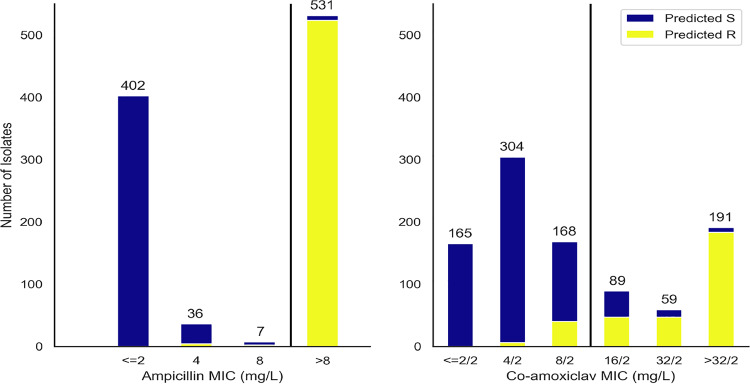
Proportion WGS predicted resistant (extended algorithm) by routine laboratory MIC.

The distribution of MICs in isolates with concordant versus discordant predictions suggested an alternative explanation (Fig. 2), with the extended algorithm performing better at predicting susceptibility/resistance in non-peri-breakpoint isolates. Overall, the algorithm correctly classified 463/469 (99%) isolates with MICs of ≤4/2 mg/liter as susceptible and 230/250 (92%) isolates with MICs of ≥32/2 mg/liter as resistant. Notably, of 79 discordant isolates containing only non-inhibitor-resistant beta-lactamases, 64 (81%) had peri-breakpoint (8/2 to 16/2 mg/liter) MICs.

Given these findings, we therefore investigated two other hypotheses that could explain the low agreement in peri-breakpoint isolates: (i) variable accuracy of the different phenotypic methods and (ii) the binary resistant/susceptible classification being too simplistic.

### Variability in reference standard agar dilution phenotypes (EUCAST and CLSI based).

A total of 291/976 (30%) isolates were selected for repeated agar dilution phenotyping using stratified random subsampling to enrich for resistant isolates both with and without beta-lactamase genes (see Materials and Methods; see also the supplementary methods and Fig. S1 in the supplemental material). Of these 291 isolates, 261 (90%) passed the additional quality control steps designed to remove potential undetected mixtures and were included in the agar-dilution subsample (details in given in the supplementary methods). In brief, all colonies had to be of one morphology on blood-agar purity plates, and MICs for each of amoxicillin and both amoxicillin-clavulanate fixed (EUCAST)/ratio (CLSI) tests had to be in essential agreement on two or more repeat tests. The stratified random sampling enriching for resistant phenotypes meant that 160/261 (61%) subsample isolates were amoxicillin-clavulanate-resistant by routine AST (see Table S1 in the supplemental material). All STs with >10 isolates in the main sample were represented, with 52 (20%), 43 (16%), and 29 (11%) isolates being ST131, ST73, and ST69, respectively, as were all resistance gene families in the main sample (see Fig. S2).

**FIG 3:**
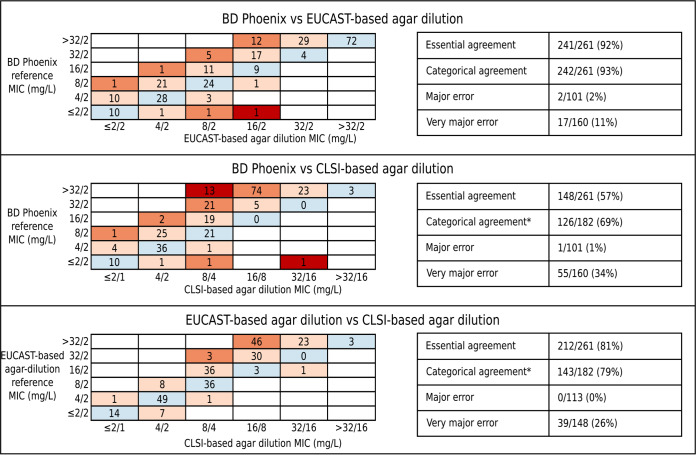
Comparison of the three different phenotyping methods on the agar dilution subsample isolates (*n* = 261). MICs obtained using three different phenotyping methods were compared: EUCAST-based agar dilution, CLSI-based agar dilution, and BD Phoenix (performed in the OUH microbiology laboratory and using panels calibrated against EUCAST guidelines). *, isolates are in categorical agreement if they are reported as either resistant by both methods (i.e., BD Phoenix/EUCAST-based agar dilution MIC > 8/2 mg/liter and CLSI-based agar dilution > 16/8 mg/liter) or susceptible by both methods (i.e., BD Phoenix/EUCAST-based agar dilution MIC ≤ 8/2 mg/liter and CLSI-based agar dilution MIC ≤ 8/4 mg/liter). Intermediate isolates were excluded from these comparisons (but are shown above) since BD Phoenix/EUCAST-based agar dilution has no intermediate category. Blue, full agreement of MICs; light orange, essential agreement; dark orange, within two doubling dilutions (theoretically feasible believing both tests having an error of ± 1 dilution); red, disagreement.

As expected, phenotypes from different reference-standard AST methods were often discordant (Fig. 3). EUCAST-based agar dilution (using the fixed 2-mg/liter clavulanate concentration) only agreed with CLSI-based agar dilution (using the 2:1 ratio of amoxicillin-clavulanate) for 143/261 (55%) isolates (27 agreed resistant, 116 agreed susceptible). For the remaining 118 isolates, EUCAST-based agar dilution results were more conservative than CLSI-based agar dilution. Major discrepancies occurred for 39 isolates, being classed resistant by EUCAST-based agar dilution and susceptible by CLSI-based agar dilution. The remaining 79 isolates were EUCAST resistant and CLSI intermediate. Excluding isolates classified as intermediate by CLSI, the categorical agreement between the two reference standard methods was 79%. Considering CLSI intermediate as resistant had little impact on the overall categorical agreement (85%). Each of these test methods also often classified isolates differently than did the BD Phoenix (Fig. 3), but, as expected, since the BD Phoenix used in the OUH routine laboratory is calibrated against EUCAST guidelines, EUCAST-based agar dilution was in agreement more often. Of note, one isolate which only contained a partial *bla*_TEM_ gene was repeatedly identified resistant on both EUCAST- and CLSI-based agar dilution testing but was identified as susceptible by the BD Phoenix.

MIC results from both methods were variable on retesting (as part of triplicate repeats): moreso for EUCAST-based agar dilution MICs (Fig. S4), which were not constant across repeats for 158/261 (61%) isolates versus only 73 (28%) for CLSI-based agar dilution MICs. Although differences across repeats were in essential agreement with one another (i.e., less than [±1 doubling dilution]) for all but 12 isolates for EUCAST-based agar dilution and all but 1 isolate for CLSI-based agar dilution, they did cause changes in resistance classification. For EUCAST-based agar dilution, 40/261 (15%) isolates were identified as both resistant and susceptible across repeats, suggesting that even within-method categorical agreement is far poorer than the standards required for regulatory approval. Likewise, for CLSI-based agar dilution, the MIC differences across repeats resulted in variation in resistance classifications for 31 (12%) isolates; however, because of the CLSI-intermediate category, 28/31 (90%) of these would be classed as minor discrepancies.

### WGS-derived resistance prediction compared to reference standard agar dilution phenotypes.

**FIG 4:**
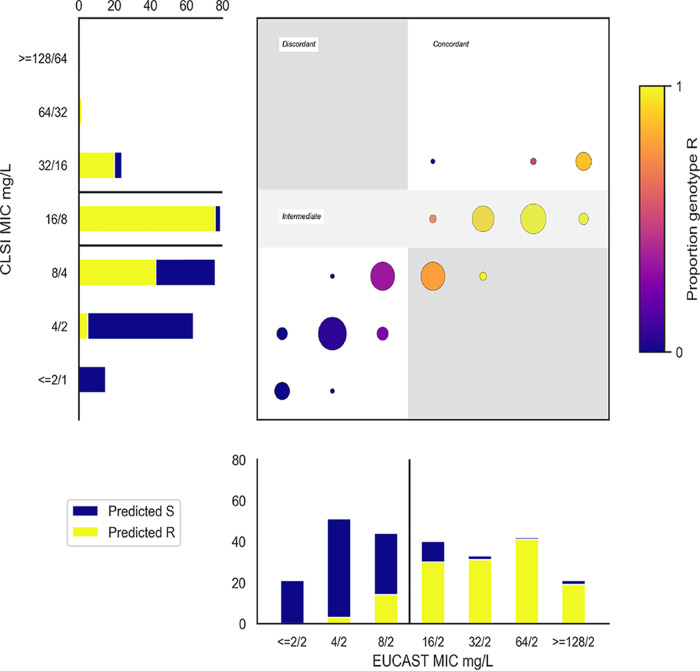
Proportion WGS predicted resistant (extended algorithm) by MICs from EUCAST and CLSI-based methods. In the main panel, each (*x* and *y*) coordinate represents (EUCAST-based MIC, CLSI-based MIC) combination. At each coordinate, the circle size represents the number of isolates with this combination of fixed and ratio MICs, and the color denotes the proportion identified as resistant by WGS, as indicated by the color bar to the right of the figure. The two subpanels (bar charts to the left and bottom of the main panel) show the number of isolates with each MIC (in line with the main panel). Yellow/blue coloring indicate which of these were predicted resistant/susceptible respectively, and black lines indicate cutoffs used to determine resistance classification (susceptible/resistant for EUCAST-based agar dilution, susceptible/intermediate/resistant for CLSI-based agar dilution).

Overall, using the extended algorithm above, WGS classified as resistant 23/27 (85%) isolates agreed resistant by EUCAST and CLSI-based tests, 107/118 (91%) indeterminate isolates (76/79 EUCAST resistant/CLSI intermediate, 31/39 EUCAST resistant/CLSI susceptible), and 17/116 (15%) agreed susceptible isolates (Fig. 4). Again, predictions based on the presence of high-copy-number (>2.5×) noninhibitor beta-lactamases alone were the least congruent with the reference standard phenotypes. Specifically, 16/62 (26%) isolates with increased copy number beta-lactamase genes were agreed susceptible (accounting for 16 of the 17 resistance predictions in agreed susceptible isolates). Further, while 46/62 (74%) isolates with this mechanism were resistant on EUCAST-based agar dilution, similar to the PPV with BD Phoenix on the whole data set, for CLSI-based agar dilution only 2/62 (3%) were CLSI resistant, 24/62 (39%) were CLSI intermediate, and 36/62 (58%) were CLSI susceptible, suggesting that this threshold performs more poorly in predicting CLSI-based agar dilution phenotypes. However, as when selecting the initial threshold against BD Phoenix results, there was no threshold which perfectly predicted the CLSI-based phenotype (see Fig. S2B in the supplemental material). Only 8/27 (30%) isolates agreed resistant by EUCAST- and CLSI-based tests contained inhibitor-resistant beta-lactamases. Conversely, 24 CLSI-intermediate and 10 CLSI-susceptible isolates contained *bla*_OXA-1_, showing that identification of inhibitor-resistant beta-lactamases was neither necessary nor sufficient to predict resistance for the CLSI-based tests.

Similarly, assessment of the individual contribution of other genetic features to the phenotype was challenging due to cooccurrence of features in the same isolate and the impact of some features on susceptibility varying both between isolates and within isolate repeats (see Fig. S3 and S4 in the supplemental material). For example, 4/9 isolates with *ampC* promoter mutations in the agar dilution subsample were both resistant and intermediate on repeat testing using CLSI-based agar dilution.

### WGS-derived resistance prediction in peri-breakpoint and non-peri-breakpoint isolates.

As with routine AST, WGS predictions of reference standard phenotypes were more accurate for non-peri-breakpoint MICs (EUCAST-based agar dilution, ≤4/2 mg/liter and ≥32/2 mg/liter; CLSI-based agar dilution, ≤4/2 mg/liter and ≥32/16 mg/liter). For EUCAST-based agar dilution, WGS correctly identified resistance/susceptibility in 169/177 (95%) isolates with non-peri-breakpoint MICs versus only 60/84 (71%) with peri-breakpoint MICs. Similarly, for CLSI-based agar dilution, excluding 79 intermediate isolates (16/8 mg/liter), WGS correctly predicted 97/106 (92%) non-peri-breakpoint isolates but predicted 43/76 (57%) isolates with MIC 8/4 mg/liter as resistant.

Interestingly, however, there were three consistently resistant (EUCAST-based MIC ≥ 32/2 mg/liter, CLSI-based MIC ≥32/16 mg/liter) and three consistently susceptible (EUCAST-based MIC ≤4/2 mg/liter, CLSI-based MIC ≤4/2 mg/liter) discrepant results. All three resistant discrepant results were explained by complexities inferring phenotype from WGS. One had a novel *bla*_CTX-M_ variant (CTX-M-15-like, Ser130Gly mutation). Previous work on mechanisms of beta-lactamase inhibition suggests mutations at Ambler position (22) 130 likely lead to inhibitor resistance (7), and a similar mutation (Ser130Thr CTX-M-190) resulted in sulbactam and tazobactam resistance (23). The other two isolates had antibiograms consistent with *ampC* hyperproduction (cefoxitin resistant, ceftazidime resistant, cefepime susceptible), but we were unable to identify complete promoter sequences matching our reference (CP009072.1) in the region upstream of *ampC.* This may suggest insertion of alternative elements upstream of *ampC* could have led to both fragmented assemblies and have driven increased expression; however, from WGS data alone it is difficult to distinguish whether this has truly occurred or instead may be due to other undetected beta-lactamase resistance mechanisms. All three isolates with susceptible discrepant results had beta-lactamases present at mildly elevated copy numbers (2.5× to 3.5× relative DNA coverage) leading to WGS prediction of resistance, which may be due to inherent unavoidable difficulty selecting cutoffs for predicting phenotype (Fig. S3B).

### Impact of individual resistance features on a continuous measure of susceptibility.

Random-effects models were used to investigate the impact of test method and WGS-identified genetic elements on agar dilution log_2_ MICs simultaneously and to create a WGS-based resistance prediction for comparison with phenotype (see the supplementary methods in the supplemental material). Elements were categorized depending on frequency (Table S5). The most predictive aspect of each element (including the presence or absence of genes and/or promoter mutations and/or gene dosage) was selected using the Akaike information criterion (AIC; see the supplementary methods). Interaction terms between genetic elements (reflecting saturation effects) and with test methodology (reflecting differential impact of the same genetic mechanism depending on the amoxicillin/clavulanate ratio) were included where *P* was <0.05.

**FIG 5:**
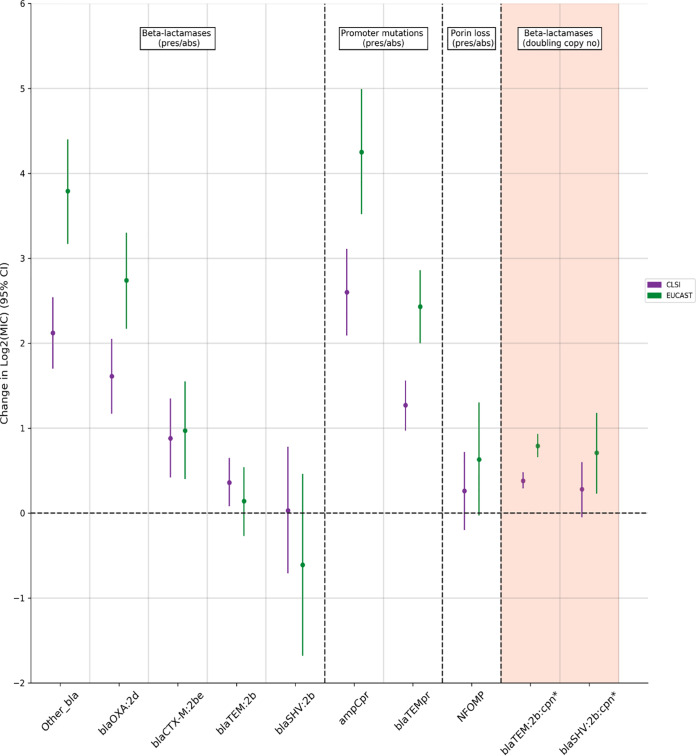
Changes in doubling dilution MIC independently associated with each feature/testing method (multivariable random-effects model). Purple represents testing using 2:1 CLSI-based agar dilution (CLSI), and green represents testing using EUCAST-based agar dilution. All elements except those denoted by an asterisk (*) and shaded in orange are modeled as binary presence versus absence effects (see the supplementary methods): other_bla (grouped other *bla* genes, includes *bla*_TEM-40_ [*n* = 2], *bla*_TEM-30_ [*n* = 3], *bla*_CMY-2_ [*n* = 3], *bla*_OXA-48_ [*n* = 1], *bla*_TEM-190_ [*n* = 1], *bla*_TEM-33_ [*n* = 1]; Table S2 in the supplemental material), blaOXA:2d (Bush-Jacoby 2d, *bla*_OXA_), blaCTXM:2be (Bush-Jacoby 2be, CTXM), blaTEM:2b (Bush-Jacoby 2b, *bla*_TEM_), blaSHV:2b, (Bush-Jacoby 2b, SHV), ampCpr (ampC promoter mutation suggesting increased expression), blaTEMpr (*bla*_TEM_ hyperproducing promoter), NFOMP (nonfunctional *ompF/ompC*), blaTEM:2b:cpn (copy number) effect modeled as effect of doubling copy number, and blaSHV:2b:cpn (copy number) effect modeled as effect of doubling copy number.

All beta-lactamases were associated with increased MICs in univariable models (see Table S6 in the supplemental material), and these associations generally persisted in multivariable models, although their magnitude decreased markedly (Fig. 5; see also Table S7). The largest effects of beta-lactamase presence were for *bla*_OXA-1_ (a class 2d beta-lactamase, denoted blaOXA:2d) and members of the “other” group of beta-lactamases, comprising either inhibitor-resistant beta-lactamases (*n* = 10), or those with unknown impact on beta-lactam susceptibility (*n* = 4) (Table S5). These caused 2- to 3-fold and 4-fold doubling dilution increases in EUCAST-based MIC, respectively. The effects of non-inhibitor-resistant *bla*_TEM_ (denoted *bla*TEM:2b) and *bla*_SHV_ (denoted blaSHV:2b) genes were more complex. For each, presence alone in an isolate was only associated with a small, often nonsignificant increase in MIC by either method [*bla*_TEM_, impact on change in log_2_(MIC) CLSI-based = +0.36 (*P* = 0.01), EUCAST-based = +0.14 [*P* = 0.51]; *bla*_SHV_, change in log_2_(MIC) CLSI-based = +0.03 (*P* = 0.93), EUCAST-based = –0.61 (*P* = 0.27)]. However, for both, a higher copy number (i.e., gene dosage) was associated with a higher MIC. These effects were small but additive [e.g., EUCAST-based MIC change in log_2_(MIC) per doubling of copy number *bla*_TEM_ = +0.79 (*P* < 0.0001), *bla*_SHV_ = +0.71 (*P* = 0.004); see details in Table S7A in the supplemental material). Like beta-lactamases, all “significant” promoter mutations were associated with increased MICs (*P* < 0.0001). In particular, “significant” *ampC* promoter mutations were independently associated with large increases in MIC [impact on change in log_2_(MIC) CLSI-based = +2.60 (*P* < 0.0001), EUCAST-based = +4.25 (*P* < 0.0001)]. Interestingly, there was no clear change in MIC independently associated with suspected porin loss in our data (*P* ≥ 0.06), despite porin loss being associated with a large effect in unadjusted analysis [see Table S6, change in log_2_(MIC) +2.28 (CLSI) and +4.17 (EUCAST)].

Of note, when increased copy number effects were included, EUCAST-based testing methodology accentuated increases in MIC caused by genetic resistance features other than for suspected porin loss and the presence of *bla*_CTX-M_ genes (the blaCTX-M:2be group; *P*_heterogeneity_ ≤ 0.05). EUCAST-based methodology however was also associated with increased between and within sample standard deviation (Table S7B).

### Predictions of MIC in an independent validation set.

**FIG 6:**
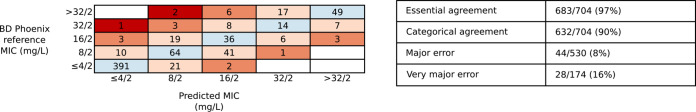
Model-based MIC prediction for non-subsample isolates (*n* = 704). Blue shading indicates correctly predicted observed AST MIC (554/704 [79%] isolates), light pink indicates predicted within one doubling dilution (total 683/704 [97%] isolates, essential agreement), orange indicates within two doubling dilutions (total 701/704 [100%]), and red indicates >2 doubling dilutions. The results shown exclude eleven isolates with resistance mechanisms not included in the agar-dilution subsample on which the prediction model was derived (similar overall performance including these).

Final EUCAST-based agar-dilution model estimates were then used to predict MICs for the 715/976 nonsubsample isolates, which were then compared to BD Phoenix MICs. MIC predictions were in agreement for 557/715 (78%) isolates and in essential agreement (within ±1 doubling dilution) for 691/715 (97%). However, these 715 nonsubsample isolates included 11 isolates that contained resistance mechanisms not present among the agar dilution subsample isolates from which the model was derived (e.g., different beta-lactamase variants). Excluding these, prediction performance was similar, with agreement for 554/704 (79%) isolates (Fig. 6) and essential agreement for 683/704 (97%) isolates. Similar to comparisons between the different antimicrobial susceptibility testing methods (Fig. 3), agreement between predicted and observed resistant/susceptible classifications was lower (90%) despite having high essential agreement of MICs. While the overall performance was good, three isolates had predicted MICs three doubling dilutions lower than observed. One had an unusual yet reproducible phenotype (ampicillin susceptible, amoxicillin-clavulanate resistant). This rare phenotype has generally been found in non-E. coli *Enterobacteriaceae* and is thought to be due to either mechanisms of *ampC* induction (24) or to the differential activity of amoxicillin and ampicillin (25). We were unable to identify a clear causative mechanism in this isolate; however, of note, it was the only isolate to contain a –11 C→T *ampC* promoter mutation. The other two both had observed MIC ≥32/2 mg/liter but only contained a low copy number *bla*_TEM-1_ and had predicted MIC 8/2 mg/liter.

## DISCUSSION

Decisions about broadening recommended empirical antimicrobial regimens from amoxicillin-clavulanate are currently being made based on unclear AST data which appears poorly concordant with WGS-identified determinants of beta-lactamase resistance. Here, we have demonstrated that this lack of concordance is not due to unknown genetic features or inherent phenotyping problems as previously hypothesized (26). Instead, it appears to arise from poor interpretation of how known genetic mechanisms of resistance impact phenotype. In contrast to the often assumed paradigm that beta-lactam resistance is generally due to the presence of specific beta-lactamases alone, mechanisms of resistance to amoxicillin-clavulanate seen regularly in a large unselected clinical data set were multifactorial, resulting from combinations of multi-copy beta-lactamase genes, mutations in resistance gene-associated promoters, and inhibitor resistance mechanisms. The individual effects of some of these features on MIC were small, variable, and additive, resulting in only minor shifts around clinical breakpoints. This potentially explains inconsistencies on repeated phenotyping which may be a consequence of the genetic basis of resistance rather than an inherent test weakness. A further corollary is that discrepancies between genotypic predictions and phenotype are inevitable when using susceptible/resistant binary classifications. Finally, the phenotypic testing methodology significantly affected the magnitude of the effect of these resistance features on the MIC. These issues, when combined, resulted in inconsistent binary phenotypes despite reliable MICs and consequently led to inevitable suboptimal concordance both between different phenotypic testing methodologies and also with WGS-based susceptibility/resistance predictions. An alternative approach would be to use WGS to predict MICs directly. We demonstrated this was possible by predicting the MIC to within one doubling dilution (essential agreement) of the observed MIC for 97% of isolates from a population-representative set of E. coli BSI.

Our study highlights the importance of isolate sampling frame, phenotyping method, and breakpoint selection. A previous study of 76 E. coli isolated from cattle (19), which reported high sensitivity and specificity of WGS to predict amoxicillin-clavulanate resistance, contained highly resistant isolates (30% containing *bla*_CMY-2_) and only attempted to predict CLSI-defined resistance (≥32/16 mg/liter). In contrast, in our study, similar to other population representative studies of human isolates with human interpretive criteria (6, 14), only a small proportion of amoxicillin-clavulanate resistance was due to inhibitor-resistant beta-lactamases, with most of the resistance being due to hyperproduction of beta-lactamases. Further, while EUCAST argue that pharmacodynamic data support choice of breakpoint and clavulanate concentration (9, 27), there is no definitive evidence as to which method has stronger associations with either clinical outcome or genotype. We therefore assessed WGS against both commonly used methods (EUCAST and CLSI).

Compared with other studies of BL/BLIs and E. coli causing human infections, we found less BL/BLI resistance was accounted for by inhibitor-resistant beta-lactamases (20). To identify resistance in our population-representative set of isolates, we found it critical to consider genetic features that alter the expression of beta-lactamases. Although the individual effects of some of these features on MICs were small, they were important because the MICs for many isolates were close to the breakpoint. Further, given the small size of these effects and effects of testing methodology, isolates could exhibit either susceptible or resistant phenotypes on repeat testing, supporting the concept of an “intermediate” phenotype, which is not accounted for in the EUCAST guidelines. The discrepancies between EUCAST and CLSI phenotypes that we observed were similar to previous studies (3), suggesting that phenotypic interpretation for one of our most commonly used clinical antibiotics remains open to question. The one mechanism for which we found no evidence of effect was porin loss: this may reflect difficulty detecting these effects from WGS alone or be a simple power issue given rarity of this mechanism in our population-based sample, since porin loss has been associated with raised MICs to other BL/BLI combination antibiotics in isolates containing Klebsiella pneumoniae carbapenemases (28).

The main study strengths are the large, population-representative sampling frame; detailed, replicated, reference-grade phenotyping for a substantial subset of isolates on which prediction models were developed (“training set”) and a large number of additional isolates with single phenotypes assessed by a commercial clinically accredited platform (“test set”), detailed and complete genotyping, and the statistical modeling. Although model performance on other data sets with geographical differences in resistance mechanisms and prevalence is unknown, its good performance in unselected clinical isolates with resistance mechanisms commonly seen in practice (in contrast to many previous studies of WGS-based resistance prediction [18]) suggest it may be generalizable. Further, the consistency of individual findings with previous literature (including inconsistent phenotype [3], mechanisms of genetic resistance [14] and poor performance of beta-lactamase presence/absence-only prediction for BL/BLI resistance [20, 29]) provide confidence that the combined results may apply across different settings. A further study strength was our use of more complete representations of the mechanisms of beta-lactam resistance. Compared to other studies of WGS-based resistance prediction which have either just used the presence of beta-lactamases or machine learning methods directly on sequence data, our models more easily align with traditional approaches of studying antimicrobial resistance and provide interpretable estimates of the direct effects of different mechanisms. A limitation is that we could only investigate proxies for some important genetic features, e.g., increased DNA copy number leading to increased expression. WGS is unable to directly quantify these effects, which thus require additional characterization by alternative methods, leading to concern that resistance prediction from WGS alone would be highly challenging. In practice, however, the good predictive performance of our model using relatively simple proxies suggests many of these features can indeed be approximated from WGS data. Modeling associations between resistance features and MIC directly allowed us to avoid inferring the phenotype from the genotype using prespecified rules and account for the effects of multiple features existing in individual isolates. The complexity of the underlying associations we discovered highlights the challenges facing standardized methods for predicting resistance across multiple drugs and species (30), and the need for automated approaches based on machine learning to take into account proxies for increased expression.

The main limitations of this study relate to its size. While we determined repeat agar dilution phenotypes for a relatively large number of isolates (*n* = 261) compared to other studies (3), many resistance elements were still rare. This had three important consequences: some infrequent features had to be categorized together for modeling, interactions between all combinations features (e.g., combinations of beta-lactamases) could not be definitively assessed, and some mechanisms present in our testing set were not present in the training set (e.g., some rare known beta-lactamase variants, many only in a single isolate [see Table S2 in the supplemental material]), and so their effect could not be estimated. Model results, however, suggested these had limited consequences. First, the features causing the greatest MIC increases were those traditionally associated with amoxicillin-clavulanate resistance (7), their specific impact being modeled here for the first time. Second, only a small number of isolates had resistance mechanisms not seen in the training data set (*n* = 11), meaning impact on performance was minimal (essentially their effect was assumed to be 0). This issue is inevitable given the substantial diversity of E. coli and incomplete knowledge of resistance mechanisms, but the excellent performance in the remaining 704/715 isolates suggests the vast majority of clinical isolates could be amenable to WGS-based MIC prediction, leaving a much smaller, more tractable number of isolates needing additional phenotypic investigation. Another potential limitation was the use of agar dilution as our reference standard phenotype, a method which, while previously endorsed by EUCAST (31), is no longer recommended, with broth microdilution now recommended instead. In contrast, CLSI still considers agar dilution as equivalent to broth microdilution (32). Reassuringly, differences we found between the BD Phoenix and agar dilution were similar to a previous study comparing BD Phoenix with reference standard broth microdilution (33), suggesting this would have relatively little impact on our overall results.

In summary, amoxicillin-clavulanate resistance in E. coli is quantitative, rather than qualitative; in reality, resistance is a continuum built up by many individual features, inevitably resulting in poor reproducibility and suboptimal concordance with binary classifications. WGS can identify the causes of amoxicillin-clavulanate resistance in E. coli provided the approach is extended to consider the complicated, polygenic, and expression-related nature of this resistance. This suggests a genetic approach could offer a less assay-dependent way to assess amoxicillin-clavulanate resistance. With renewed interest in using BL/BLIs to treat highly drug-resistant infections, our study has implications for both clinical practice and research. Given amoxicillin-clavulanate susceptibility phenotypes are variable and highly dependent on the phenotypic method used, they must be interpreted with caution. Genetic approaches have the potential to circumvent this issue, although the relationship between different resistance mechanisms and clinical outcome is not yet fully understood. Further, our findings challenge the assumption that amoxicillin-clavulanate resistance is binary (susceptible/resistant) since the same underlying resistance feature can be associated with MICs just below or just above the breakpoint. Given the variability and complexity in both the underlying mechanisms and resulting phenotype, a more transparent approach considering background genetic features, the expression levels of beta-lactamases, MIC values, and the clinical syndrome is likely needed to guide management decisions.

## MATERIALS AND METHODS

### Study population and routine microbiological processing.

E. coli isolated from all monomicrobial or polymicrobial blood cultures at OUH NHS Foundation Trust between 1 January 2013 and 31 August 2015 were included, excluding repeat positive cultures within 90 days of an index positive. Automated AST was performed in the routine laboratory (BD Phoenix; Becton, Dickinson and Company), and MICs were interpreted according to EUCAST breakpoints. Data were extracted from the Infectious Diseases in Oxfordshire Research Database (34), which has Research Ethics Committee and Health Research Authority approvals [14/SC/1069, ECC5-017(A)/2009].

### DNA extraction and sequencing.

Isolates were recultured from frozen stocks stored in nutrient broth plus 10% glycerol at –80°C. DNA was extracted using the QuickGene DNA Tissue Kit S (Kurabo Industries, Japan) according to the manufacturer’s instructions, with an additional mechanical lysis step (FastPrep; MP Biomedicals) immediately after chemical lysis. A combination of standard Illumina and in-house protocols were used to produce multiplexed paired-end libraries which were sequenced on the Illumina HiSeq 2500, generating 151-bp paired-end reads. High quality sequences (see the supplementary methods) were *de novo* assembled using Velvet (35) as previously described (36). *In silico* Achtman (37) multilocus sequence types (MLST) types were defined using ARIBA (21).

### Evaluating the importance of genetic features that modify effective beta-lactamase concentration.

We identified components of two genetic resistance prediction algorithms for amoxicillin and amoxicillin-clavulanate (Table 2; see also the supplementary methods in the supplemental material) using ARIBA (21) (default parameters) and tBLASTn/BLASTn (38). The “basic” prediction used only presence/absence of relevant genes in the Resfinder (17) database, and the “extended” prediction additionally included *bla*_TEM_ and *ampC* promoter mutations, estimates of DNA copy number, and predicted porin loss of function. A high DNA copy number was used as an indicator of possible gene duplication or high plasmid copy number, which are both known to cause increased beta-lactam resistance (39, 40). We made no attempt to distinguish between these two causes due to the limitations of short-read sequencing data. For *bla*_TEM_ and *ampC* promoters, sequences identified using ARIBA/BLASTn were searched for variant sites and regions previously associated with significantly increased expression (41–43). For transmissible resistance genes, we estimated DNA copy number by comparing mapping coverage with the mean coverage of MLST genes and defined a relative coverage of >2.5 as increased copy number (based on receiver-operator-curve [ROC] analysis; see the supplementary methods and Fig. S3A in the supplemental material). Finally, sequences found by ARIBA using reference *ompC* and *ompF* sequences (RefSeq no. NC_000913.3) were inspected for features such as indels and truncations suggesting functional porin loss.

### Evaluating the impact of different phenotypic methods.

A subset of 291 isolates were selected for replicate agar dilution phenotyping using random sampling within strata defined by phenotype-genotype combinations (see the supplementary methods for full subsampling procedure; see also Fig. S1A and B). Replicate agar dilution phenotyping used clavulanate concentration and MIC interpretation according to both EUCAST and CLSI guidelines. The aim was to explore reasons for discordance between observed phenotype and predictions made on the basis of beta-lactamase gene presence or absence alone. The subsampling therefore aimed to enrich for several groups of isolates, including resistant (by BD Phoenix) isolates both with and without beta-lactamases identified from WGS, isolates with peri-breakpoint MICs and susceptible isolates containing beta-lactamases. For each method, subcultures (from frozen stocks) were tested in triplicate using ISO-Sensitest agar plates containing amoxicillin and clavulanate in a 2:1 ratio (CLSI) or a fixed concentration of clavulanate (2 mg/liter; EUCAST), with E. coli controls ATCC 25922 (wild type) and ATCC 35218 (TEM-1 beta-lactamase producer) (44). For additional quality control, bacterial isolates were plated on sheep blood agar and incubated overnight at 37°C to check purity, with isolates excluded if multiple colonial morphologies were seen. Isolates were included in analyses if two or more MICs for each of amoxicillin, EUCAST-based amoxicillin-clavulanate and CLSI-based amoxicillin-clavulanate were in essential agreement (i.e., a minimum of 2/3 for each drug; see the supplementary methods). Isolates with less than two MICs for each of the tests passing quality control were tested an additional time to reduce the risk of selection bias against isolates with genetic mechanisms causing variable expression and underestimating natural phenotypic variability. For each included isolate, susceptibility classification for that isolate was defined using the “upper median” MIC (choosing the higher MIC when the median lay between two MIC readings) of the test repeats.

### Modeling and predicting MICs.

Random-effects models (Stata 14.2; StataCorp LP) were used to investigate the impact of test method and WGS-identified genetic elements on agar dilution log_2_ MICs simultaneously (for additional details, see the supplementary methods). Elements were categorized depending on frequency (Table S4). Models included method-specific random effects for each isolate and testing batch and method-specific (heteroskedastic) errors. All genetic element categories were included *a priori*, but the most predictive effects of each (including presence or absence of genes and/or promoter mutations and/or gene dosage) were selected using the Akaike Information Criterion (AIC; see the supplementary methods). Lastly, interaction terms between genetic elements (reflecting saturation effects) and with test methodology (reflecting differential impact of the same genetic mechanism depending on the amoxicillin/clavulanate ratio) were included where *P* was <0.05. Final estimates were then used to predict MICs in all nonsubsample isolates and in nonsubsample isolates which did not contain resistance features not present in the agar dilution subsample. Predicted MICs were then compared to routine laboratory phenotypes from the BD Phoenix.

### Data availability.

Sequences used in the study are made available at PRJNA540750. MIC data and code used for this analysis are available at https://github.com/TimothyJDavies/reconciling_the_potentially_irreconcilable.

## Supplementary Material

Supplemental file 1
